# Sugar Content and Sources in Commercial Infant Cereals in Spain

**DOI:** 10.3390/children9010115

**Published:** 2022-01-17

**Authors:** Liliana Garro-Mellado, Eduardo Guerra-Hernández, Belén García-Villanova

**Affiliations:** Department of Nutrition and Food Science, Faculty of Pharmacy, University of Granada, 18071 Granada, Spain; lilianagarromellado@gmail.com (L.G.-M.); ejguerra@ugr.es (E.G.-H.)

**Keywords:** complementary feeding, porridge ingredients, sugars determination

## Abstract

Instant infant cereals reconstituted with infant formula are the first complementary food for most Spanish infants. The main aim is to provide information on sugars in the formulation of infant cereals. Product information was collected from department stores, supermarkets, and pharmacies and completed with data from brand websites. A portion of the samples was selected for total sugars determination using the HPLC and Luff-Schoorl methods. The information regarding a total of 120 milk-free instant infant cereals marketed in Spain from 12 companies was summarized. The mean of total sugars was 23 ± 9 g/100 g (25–42%), providing 24% of the calories. Most of porridges are prepared with partially hydrolyzed flours providing free sugars (glucose and maltose). The most commonly added sugar is sucrose. A total of 43.3% of products contain added sucrose, and 16.7% contain fruits. Infant cereals analyzed with added sugars can have a sugar content similar to that found in products without added sugars. Consistent differences were found in sugars content between assayed methods and this label information. Although the European legislation of infant cereals establishes values for added sugars, the labeling reflects the content of total sugars, but not that of added sugars.

## 1. Introduction

According to the World Health Organization (WHO), the adequate nutrition of infants is essential to ensure the growth and optimal development of children and achieve better health throughout life, including prevention of overweight, obesity, and diet-related non-communicable diseases [1]. The WHO and the Nutrition Committee for the European Society for Paediatric, Gastroenterology, Hepatology, and Nutrition (ESPGHAN) recommend exclusive breastfeeding for the first six months of life [2].

The transition period from exclusive breastfeeding to family foods is referred to as complementary feeding and usually covers the period from 6 to 18–24 months of age. The ESPGHAN recommends that the complementary feeding (i.e., solid foods and liquids other than breast milk or infant formula and follow-on formula) should not be introduced before 17 weeks and not later than 26 weeks [2], and this recommendation is supported by the European Food Safety Authority (EFSA) which concludes that four to six months is a safe period [3].

Complementary feeding, also called weaning, includes different types of food, i.e., vegetables, fruits, starchy foods such as cereals and potatoes, meat, fish, and dairy products. There are no harmonized approaches to food that should be introduced in different countries [4]. International and National recommendations and guidelines have been developed to guide healthcare professionals and parents regarding the transition from milk to foods [5].

The European Commission defines infant cereals as “processed cereal-based foods” that are divided into “simple cereals which are or have to be reconstituted with milk or other appropriate nutritious liquids” or “cereals with an added high protein food which are or have to be reconstituted with water or another protein-free liquid”. Infant cereal porridges are an important energy source for the nutrition of infants in Mediterranean countries, and they constitute the basis of their complementary feeding from the age of 4–6 months. Historically, the Spanish infant population was given foods based on cereals, processed at home by toasting and boiling cereal flours. Currently, infant cereals are processed by dietetic product manufacturers in large-scale factories. These products undergo toasting or boiling and a hydrolysis and drying process to improve their sensory qualities, digestibility, safety, and shelf-life [6].

Cereal porridge reconstituted with milk is the first food introduced in complementary feeding in most Spanish infants. These are consumed up to at least two years old. Other ingredients optionally added to instant cereal porridges include sucrose, glucose or fructose, honey, powdered fruit, powdered milk, minerals, vitamins, and flavors. Among all of the present sugars, glucose, fructose, maltose, lactose, and sucrose are the most relevant nutritionally [7]. The sugars present in infant cereals may come from different sources, i.e., cereal hydrolysis, direct addition of ingredients such as sugar, fruit, honey, cocoa, and addition of milk to reconstitute them.

Current European legislation allows adding sugars to processed cereal-based food and infant food for infants and young children. It states sucrose, fructose, glucose syrups, or honey (i.e., simple cereals which are or have to be reconstituted with milk or other appropriate nutritive liquid). The amount of added sugars from these sources should not exceed 7.5 g/100 kcal and 3.75 g/100 kcal for fructose [8]. The WHO recommends [9] that “free sugars” should not provide more than 10% of total energy intake and suggests limiting them to 5% of energy would have additional benefits in reducing the risk of non-communicable diseases (specifically weight gain and dental caries). The ESPGHAN recommends an even lower intake of free sugars for infants and toddlers under two years old [10], and the American Health Association recommends to avoid added sugars for children under two years old [11]. This recommendation aims to decrease cardiovascular disease risk among children (excess weight gain and obesity, elevated blood pressure and uric acid levels, dyslipidemia, and non-alcoholic fatty liver disease), insulin resistance, and type 2 diabetes mellitus (T2D) and also to maintain diet quality. This is of concern, because no nutritional requirements have been established regarding free sugars, and infants have an innate preference for sweet taste, which may be modified and reinforced by pre- and postnatal exposures [5].

The main aim is to provide information on sugars in the formulation of infant cereals, determining their type and quantity, estimating their origin and evaluating the percentages of their contribution to energy in infants.

## 2. Materials and Methods

### 2.1. Samples and Information Selected

A total of 120 samples of commercial infant cereal porridges (dry) were included in this study. The products were defined as food lines marketed for complementary feeding, predominantly for infants from 4 to 12 months.

A comprehensive search between April and August 2017 was performed by accessing the leading supermarket websites, drugstores websites, and physical stores (local supermarkets, pharmacies, etc.) in the Granada area to know the infant cereal marketed in Spain. The information about the products was completed and confirmed with the data found on the brand websites. Twelve companies market these products in Spain, and each brand produces between 2 and 32 different infant cereal porridges (powdered). The brands of products found were the followings: Nestlé, Almirón, Hero infant, Hipp, Puleva, Nutribén, Blevit, Sanutri, Infantbio, Damira, HB Pedialac, and Milupa.

A database was created using the information reported on the labels of commercial infant cereals porridges as following: brand, product denomination, ingredients, elaboration process, nutritional values, nutritional benefits, dosage, and recommended age.

### 2.2. Sample Selection for Sugars Analysis

Twelve samples (10% of all porridges) were selected to analyze the sugar content in infant cereals. The following criteria were considered to select the samples:

- 8 Cereals common factor: all selected infant cereals were “8 cereals”, because this product was in most brands.
- With sugars (sucrose and honey) or without added sugars (according to the label information): samples with added sugars (samples A, C–F, and K), and others without added sugars (samples B, G–J, and L).
- With other ingredients with natural sugars: samples F and L contained powdered fruits.

According to the label information, the flours from samples A–C, F, I, and K were enzymatically hydrolyzed. The flours of sample J were thermally hydrolyzed. However, in the rest of the samples, this information was not included on the label (D, E, G, H, and L); instead, the list of ingredients specifies “hydrolyzed” or “partially hydrolyzed” flours. The hydrolysis process increased the sugar content of infant cereal porridges.

#### Ingredients of Analyzed Samples

Sample A—“8 cereals and fructooligosaccharides (FOS)”: partially hydrolyzed cereal flours 87.7% (wheat, corn, rice, oat, barley, rye, buckwheat, and millet), sucrose, and FOS.

Sample B—“8 cereals”: dextrinated cereal flours 92% (wheat, rice, barley, rye, corn, millet, sorghum, and oat), FOS 3%, malt extract, and maltodextrin.

Sample C—“8 cereals with gluten”: hydrolyzed flours of 8 cereals 86% (wheat, corn, rice, oats, barley, rye, sorghum, and millet), sucrose, and maltodextrin.

Sample D—“8 cereals and FOS”: hydrolyzed cereals 91% (wheat, corn, rice, oat, barley, rye, sorghum, and millet), sucrose, FOS 3%, and malt extract.

Sample E—“8 cereals, honey and FOS”: partially hydrolyzed cereal 76.9% (wheat, corn, rice, millet, oat, barley, rye, and buckwheat), sucrose, honey powder 8%, and FOS.

Sample F—“8 cereals with fruits and FOS”: partially hydrolyzed cereal flours (79.8%) wheat, corn, rice, millet, oat, barley, rye, and buckwheat), sucrose, FOS, fruit flakes (2.1%; apricot, banana, and orange).

Sample G—“Multigrain, (8 cereals)”: hydrolyzed 8 cereals 99% (wheat, whole grain wheat (30%), corn, rice, oat, barley, rye, sorghum, and millet).

Sample H—“8 cereals”: hydrolyzed flours of 8 cereals 99% (whole grain wheat (46%), wheat, corn, rice, oat, barley, rye, sorghum, and millet).

Sample I—“8 cereals”: flours 97.1% (hydrolyzed wheat, wheat, barley, rye, corn, rice, millet, sorghum, and oat), oligofructose, inulin, and bifidobacteria. 

Sample J—“8 cereals”: cereal flours 99% (wheat, barley, rice, oat, rye, millet, and sorghum), maltodextrin, and corn starch.

Sample K—“8 cereals with honey”: hydrolyzed flours of 8 cereals (whole grain wheat (50%), wheat, corn, rice, oat, barley, rye, sorghum, and millet) and honey (6%).

Sample L—“8 cereals with fruits”: hydrolyzed flour of 8 cereals 96% (wheat, whole grain wheat, (30%), corn, rice, oat, barley, rye, sorghum, and millet), and dehydrated fruits 3% (banana, apple, and orange).

A pool of vitamins and minerals was added to all cereal porridges, and some products included flavorings.

### 2.3. Individual Sugars Determination by HPLC

Sugar content (glucose, fructose, sucrose, maltose, and lactose) was determined by HPLC.

#### 2.3.1. Sugar Extraction Procedures

An adaptation of the method developed by Hernández et al. was performed for the sugar extraction of infant cereal samples [12].

Firstly, 0.05 g of each sample was added to a 15 mL centrifuge tube with 4 ml of distilled water (Milli-Q^®^ Ultrapure Water System; Millipore Co., Burlington, MA, USA). The tubes were shaken for 1 min and were placed in an ultrasonic bath (J.P. Selecta, Barcelona, Spain) to sonicate for 15 min and subsequently placed into a water bath (GFL, Burgwedel, Germany) at 50 °C for 30 min with shaking at 50 rpm. Then, the tubes were cooled and centrifuged (Hettich, Tuttlingen, Germany) at 9000 rpm for 10 min. This procedure was repeated three times. The supernatants of each centrifugation were collected in another tube and were clarified using 0.25 mL of Carrez I (15% potassium ferrocyanide) and 0.25 mL of Carrez II (30% zinc acetate) reagents (Merck, Darmstadt, Germany), shaken for 1 min and centrifuged at 9000 rpm for 10 min. The clarified supernatant was transferred into a 25 mL graduated flask and made up to volume with distilled water. Two milliliters of this solution were filtered by a 0.2 µm filter (Millipore Corp., Burlington, MA, USA).

#### 2.3.2. Sugar Determination

The sugar contents (glucose, fructose, sucrose, maltose, and lactose) present in the samples were determined using a 940 Professional Ion Chromatograph Vario 2 (Metrohm, Herisau, Switzerland) with amperometric detection and MagICNet^TM^ software to assess the working conditions. A Metrosep Carb 2- 250/4.0 (250 × 4.0 mm) column (Metrohm, Herisau, Switzerland) with alkaline eluent (300 mM sodium hydroxide and 100 mM sodium acetate) was used. The temperature was 30 °C, and the flow rate of the eluent was 0.5 mL/min. An external standard method was used for quantification. The concentration of sugars (glucose, fructose, sucrose, maltose, and lactose) ranged from 0.5 to 50 mg/L. A linear response was obtained, and the correlation coefficients were as following: glucose (r^2^ = 0.99998), fructose (r^2^ = 0.99998), sucrose (r^2^ = 0.9999), maltose (r^2^ = 0.9999), and lactose (r^2^ = 0.9999). The results were expressed in percentages to compare the total sugars content analyzed with the information listed on the labels.

### 2.4. Total Sugars Determination by the Luff-Schoorl Method

The contents of reducing sugars and total sugars after inversion were determined using the Luff-Schoorl method [13].

#### 2.4.1. Sugar Extraction Procedures

First, 0.5 g of sample was added into a 15 mL centrifuge tube with 5 mL of 80% ethanol (*v*/*v*). This tube was shaken and sonicated for 15 min and then was taken to a water bath at 70 °C for 30 min and shaken at 50 rpm. Subsequently, it was cooled and centrifuged at 9000 rpm for 10 min. The procedure was repeated. The supernatants of both extractions were collected in another tube, which was clarified using 0.25 mL of Carrez I and 0.25 mL of Carrez II reagents, shaken and centrifuged.

#### 2.4.2. Sugar Determination (Reducing Sugars and Total Sugars)

For reducing sugars, 5 mL of the extraction were placed with 5 mL of distilled water in an Erlenmeyer flask with exactly 10 mL of Luff-Schoorl reagent. This solution was heated at 100 °C for 5 min and then cooled in an ice bath. After 5 min, 10 mL of a 30% potassium iodide solution, 10 mL of a 10 N sulphuric acid solution, and 3–4 drops of a starch solution were added to the mixture; then, reducing sugars were determined by titration with 0.05 N sodium thiosulphate. A blank assay must be performed. The sugar determination was performed using a calibration curve for glucose with aliquots from 5 to 24 mg/mL (R^2^ = 0.9985).

To determine total sugars, few drops of a 0.1% (*w*/*v*) methyl orange solution were added to 10 mL of the extraction solution. Then, several drops of a 4 N HCl solution were added, until the color changed to red, and 3 mL of a 0.1 N HCl solution were added. After that, the mixture was heated at 100 °C for 30 min and cooled in an ice bath before adding 3 mL of 0.125 N NaOH until the change of color. This solution was placed in a 25 mL graduated flask and made up to volume with distilled water. After the acid hydrolysis, the same titration procedure as in reducing sugars determination was applied. The results were expressed in percentages.

### 2.5. Estimated Energy Intake Provided by Sugars

The energy intake provided by sugars depending on the infant cereal and the age was estimated according to the results obtained by the HPLC method. The dietary references intakes (DRIs) were considered for calculating the estimated energy requirement (EER) [14]. Serving amounts were as following: 33.2 g of infant cereal, 659 kcal/day (4–6 months), and 839 kcal/day (7–12 months).

### 2.6. Theorical Sweetening Power

A theoretical sweetening power was established considering the content of each sugar and its sweetening power, taking as a reference the values provided by Guerra-Hernandez and Plaza Díaz [15]. The values applied were 0.7, 1.2, 1, and 0.55 for glucose, fructose, sucrose, and maltose, respectively.

### 2.7. Statistical Analysis

The assays of each sample were conducted in duplicate, and each sample was injected twice. The results are expressed as mean ± standard deviation (SD). The Shapiro–Wilk test was applied to determine the normality of the analyzed variables. The relationships between the different assays were evaluated by computing the relevant correlation coefficient (Pearson’s linear correlation) at the *p* < 0.05 confidence level.

The Bland and Altman method was used to assess the difference in sugars content determined by the HPLC and Luff-Schoorl methods at the *p* < 0.05 confidence level. In addition, the sugar contents in the analyzed samples were compared with the sugar contents provided on the labels of the selected samples (*n* = 12), according to the information registered in the aforementioned database, by using the Bland and Altman method.

All statistical analyses were performed using IBM SPSS Statistics for Windows, version 26 [16].

## 3. Results

### 3.1. Nutritional Information Labeling

A total of 120 products named “infant cereal porridges” of 12 brands marketed in Spain were included in this study. The average nutritional values per 100 g of the product for 120 samples were as following: energy, 382 kcal; lipids, 1.8 g; carbohydrates, 81 g; sugars, 23 g; protein, 8.2 g; and fiber, 4.7 g; the ranges of values were large for all nutrients (Table 1).

### 3.2. Ingredients of Infant Cereal Porridges

Table 2 shows the different brands and the numbers of products, including ingredients others than cereal flours.

According to the information declared on the labels of infant cereals, the products are classified with (72.5%) or without gluten (27.5%). Gluten-free infant cereals contain rice flour, or rice and corn flours, instead of the samples containing gluten that mainly contain wheat, barley and rye flours. All brands market gluten-free infant cereals.

Most products include cereal flours such as wheat, rice, corn, oat, rye, barley, millet, and sorghum, but the same brands include pseudocereals, i.e., buckwheat or quinoa. The information of ingredients listed on the label regarding cereal flours states “hydrolyzed flours” for some products, or the hydrolysis process of the flours is collected somewhere on the label. Both types of information were not collected for all products included in this study. Other carbohydrate sources, such as starch of corn and barley, malt and maltodextrin as well as yucca and carob tree flours are included in the formulation of some products.

In the manufacture of infant cereals, hydrolyzed flours or hydrolysis of cereals increase the sugar contents (glucose and maltose). The total sugars collected in the nutritional information (labeled) include natural sugars of the flours and those produced during the processing of porridges, in addition to those from other ingredients.

Commonly added sugars are sucrose, and some brands also add glucose, brown sugar, and honey or cane honey in some products. Ten out of the 12 brands use sucrose in their formulation, but it is only present in 43.3% of commercialized infant cereals. Glucose and brown sugar are only included in some products. Honey is also commonly added to cereals to improve the sensory properties and provide a sweet taste. The main honey sugars are glucose and fructose. Only 2 out of the 12 assessed brands do not use honey in some of their products; the rest have some products with honey providing high sensory quality.

The fruits added to infant cereal products are apple, pear, banana, orange, pineapple, apricot, kiwi, grapes, raisins, and prunes. Fruits are added to 16.7% of infant cereals in the form of dehydrated, concentrated, or dried fruits. Fruits commonly added are apple, banana, and orange.

Oligofructose, inulin, and FOS are added by 49.1% of products. Seven out of 120 products include cocoa or chocolate powder (5.8%), and some products have nuts (almonds, hazelnuts, and walnuts) and probiotics such as bifidobacteria or lactobacillus (16.7%).

Fats are not commonly used in the formulation of infant cereals, but some products include palm, nabine, coconut, or sunflower oils.

Table 3 shows the total sugars declared on the labeling. The average sugar content in labels was 23.9 g/100 g of products. Nine out of 12 brands have an average of total sugars higher than 20%, and around 20% of products contain more than 30% of total sugars. As can be seen, more than half of products (54.3%) contain added sugars, and 38.6% of all products contain added ingredients providing natural sugars.

### 3.3. Sugar Content Determination

#### Sugar Content by the HPLC Method

Table 4 shows the total sugars content and the contents of individual sugars. Samples A, B, E, F, and K exhibited the higher contents. The average sugar content was 18.1 ± 4.55. Half of the samples had glucose contents higher than the content in other sugars. The range of glucose was 0.28–19.83%. Half of the samples contained values of maltose superior to those of glucose. The maltose values ranged between 1.73% and 14.85%.

The fructose values for most samples were below 1% (0.06–0.95%), except in samples K (“8 cereals and honey”) with a fructose value of 3.4% and sample L (“8 cereals and fruits”) with a fructose value of 1.14%. Both samples showed high values of glucose and fructose due to the fact they contained honey or fruit but not sucrose added as an ingredient.

In samples A and C–F, the sucrose values were higher than in the rest of the samples, because they contained sucrose added among their ingredients. The values ranged between 6.09 and 9.87 g/100 g. The samples without added sugars had values of sucrose between 0.15 and 1.08 g/100 g; among those, the highest value (1.08%) corresponded to the sample with added fruit. The sucrose contents in samples B, G, and H–K ranged between 0.15% and 0.38% (naturally occurring sugar); sucrose is not declared in the list of ingredients. The added sugars represented between 27% and 61% of total sugars determined.

The lactose content was below the detection limit for all analyzed samples, which confirmed the absence of milk or dairy products.

In previous studies performed by our group, intrinsic sugars “8 infant cereal” flours before processing (without other ingredients added) were determined, and 1.1% of glucose, fructose, maltose, and sucrose were quantified [14].

Table 4 shows that the difference between the sugar content labeled and the sugar content analyzed was considerable and statistically significant (*p* < 0.05), noting the biggest difference in sample K and the least difference in sample A. The higher sugar content declared on the label could be due to the lack of specificity of a commonly applied volumetric method.

As Table 4 shows, the total sugars content values obtained by the Luff-Schoorl method were higher than 24% in samples A, B, E, F, I, and K, with the average value of 22.5 ± 4.82. The sucrose content was obtained by the difference between total sugars and reducing sugars analyzed. The highest values of sucrose were found in samples A and C–F.

The differences between the sugar content labeled and the sugar content determined by this method were small in almost all samples (0.1–3.3%), except in samples J (7.66%) and K (9.07%), but no statistically significant differences were found.

### 3.4. Estimated Energy Intake Provided by Sugars

The energy intake provided by sugars depending on the infant cereal and the age was estimated according to the results obtained by the HPLC method. The Dietary References Intakes (DRIs) were considered for calculating the EER [17]. Serving amounts were as following: 33.2 g of infant cereal, 659 kcal/day (4–6 months), and 839 kcal/day (7–12 months).

**Figure 1:**
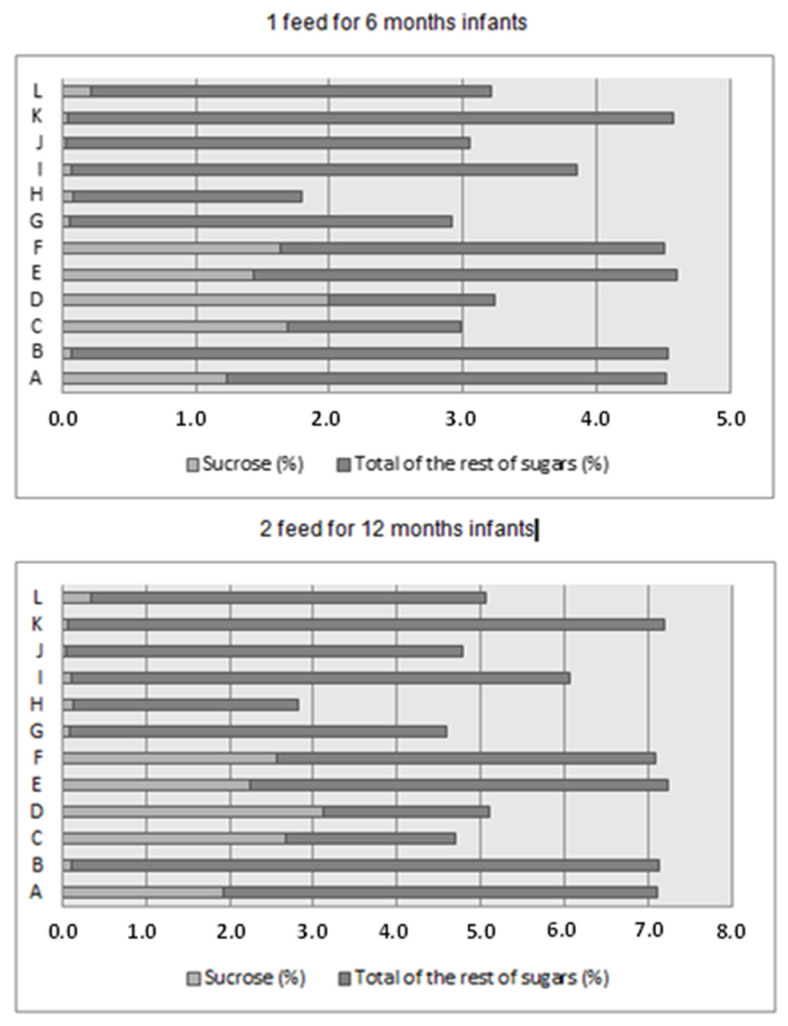
Estimated energy intake from the sugars of the infant cereals analyzed according to each sample and the age of the infants (6 and 12 months).

Figure 1 shows the percentage of calories from sucrose and the total sugar of each commercial infant cereal analyzed, assuming one meal or serving for 6 months old and two meals for 12 months old. Five samples showed approximately 4.5% of total energy provided by sugars at six months and approximately 7% at the age of 12 months. Another five samples provided approximately 3% or 5% of total energy at 6 and 12 months old, respectively, and one commercial infant cereal (sample H) had below 2% or 3% of total calories from sugars at 6 or 12 months of age, respectively.

The dose of baby cereal porridge is 33.2 g. The EERs according to the Food and Nutrition Board (FNB), Institute of Medicine (IOM 2002) from the USA are 659 kcal/day for 4–6 months and 839 kcal/day for 7–12 months.

## 4. Discussion

Cereal porridges are a good choice for the beginning of complementary feeding and even the first years of life [18], and these are the first complementary food for Spanish infants. The three main sources of sugars in infant cereals are as following: added sugars, sugars from ingredients (intrinsic sugars), and sugars produced by enzymatic hydrolysis of starches during the processing of infant cereals. The use of sucrose as an ingredient could reduce the nutritional quality of these foods. This study found that half of infant porridges had added sugars.

### 4.1. Ingredients of Infant Cereal Porridges (Dry)

Wheat and rice flours are the main cereals used in infant cereals, depending on whether porridges contain gluten (72.5%) or are gluten-free (27.5%). Four out of 12 brands included some products with whole-grain flours; complementary cereals marketed in Germany have shown that half of the products are formulated with whole-grain flours [19]. Most brands state in labels that cereals suffer a hydrolysis process; however, label information does not clarify whether companies hydrolyze the cereals in the manufacturing process or if they purchase hydrolyzed cereals. Other flours included in the formulation are maize, oat, rye, barley, millet, sorghum, triticale, spelt, quinoa, buckwheat, yucca, soy, peanut, carob, and sesame. Some infant cereals have yucca, maize starch, or maltodextrins. Soluble fiber (inulin, FOS, and oligofructose) and fruits are added by 10 of 12 brands, and only two brands contain probiotics (*Bifidobacteria* and *Lactobacillus lactis*). Half of the brands have cocoa or chocolate, and only some products contain nuts. Five brands include sugars in all products, and only one brand does not include them. The percentage of sugars included by brands range between 0.2% and 41.7%. Most infant cereals marketed in Spain are enzymatically hydrolyzed to improve the dispersibility and reduce the syneresis of products after preparation and increase starch digestibility [18]. The activity of pancreatic α-amylase is full between 12 and 18 months of age, so that in the first month α-glucosidase (maltase) and salivary α-amylase complement the pancreatic α-amylase activity [11,20], and the non-hydrolyzed starch is fermented by gut microbiome which allows recovering energy. According to the current knowledge, it could be considered that hydrolysis is an unnecessary step in the manufacturing of infant cereals [18]. Eighty-two percentage of infant cereals marketed in Spain (11 brands) are formulated with hydrolyzed flours, and only one brand with a large number of products uses non-hydrolyzed flours but introduces corn maltodextrins in the formulation.

As mentioned above, more than half of the evaluated products contained added sugars in different forms (sucrose, brown sugar, glucose, and honey), and the average sugar content was 23.2 g/100 g, being around 24% of the total calories of infant cereals. The sucrose was the most used sweetener, similar to those found in German infant cereals [19]. Maalouf et al. [21] evaluated the sugar content stated on the label of the USA commercial instant infant cereals (dry) and found an average value of 12 g/100 g, and 12% of products have one source of added sugars. However, this study identified numerous types of added sugars when compared with our study (sugar, sweetener, syrup, corn syrup, high fructose corn syrup, honey, fructose, malt, maltose, molasses, glucose, lactose, sucrose, brown sugar, and trehalose). Elliot and Conlon [22] examined the nutrition labeling of 240 packaged baby foods in Canada, noting that 45% of products have 20% of calories from sugars and approximately 29% of these products contain added sugars in the first four ingredients listed on the label. García et al. [23] evaluated the sugar contents in different commercial baby foods from the UK, finding that sweet foods have a higher content of sugars than savory foods; these findings also revealed that dry finger foods and snacks for infants have a larger sugar content (19.5 g/100 g) and are similar to our findings, with the sugar content being higher in sweet snacks containing fruits (31.8 g/100 g).

A European study on baby food (2634 products) [24] from 10 countries, including Spain, evaluated the sugar content indicated on the label; 255 (9.7%) products are instant infant cereals (dry); 74 of them (29%) come from seven brands marketed in Spain. The average sugar content reported is 21.9%, similar to our findings (23.9%); the energy percentage from total sugars ranged between 4% and 23%; Baby foods in Spain showed the highest percentage.

The EU Directive 2006/125/EC on processed cereal-based foods and baby foods for infants and young children has established that the amount of sugars (sucrose, fructose, glucose, glucose syrups, or honey) added to infant cereals should not exceed 7.5 g/100 kcal and the amount of added fructose should not exceed 3.75 g/100 kcal [25]. In this way, the legislation only limits the addition of sugars, allowing the increase in total sugars using other ingredients with natural sugar content (fruits, milk, cocoa, malt extract, etc.) or sugars produced in the manufacturing process (hydrolysis of starch). This study, according to the ingredients list, found that 43.3% (10 out of 12 brands) of infant cereals add sucrose, 8.3% add glucose (6 out of 12 brands), and 15.8% add honey (10 out of 12 brands); fructose was not added to any porridge, and this sweetener is unusual in Spanish infant food.

The Pan American Health Organization Nutrient Profile Model has established a criterion for identifying processed and ultra-processed products with excessive free sugars, estimating free sugars based on the amount of total sugars declared on food [26]. The criterion applied to excessive free sugars is that the amount of energy (kcal) from free sugars is equal to or higher than 10% of the total energy (kcal) in any given quantity of the product. Fifty-five percentage of infant cereals evaluated in this study, without added fruits (98 samples), exhibited between 20% and 30% of calories from free sugars, 15% of calories from between 10% and 20% of free sugars, 16.3% of calories from 30% and 40% of free sugars, and 3% of calories from more than 40% of free sugars. Only 9% of the samples have shown 10% of calories from free sugars. According to these results, this group of foods reports excessive free sugars [26]. The infant cereals without and with added sugars revealed between 19.3% and 30.6% of their calories, respectively, were from free sugars.

The composition and information requirements for processed cereals-based foods are regulated by Regulation (EU) n° 609/2013 [8]. According to this regulation, the EU must adopt a delegated act for these products, which is pending adoption. In 2015, the European Parliament rejected the proposal on the ground that the regulation delegated did not foresee sufficient measures for the obesity control in infants and young children, and the maximum level of sugar should be reduced according to the WHO recommendations [27].

### 4.2. Sugars Content Determination by HPLC

The glucose content analyzed by HPLC showed higher values in samples without added sugars. The highest glucose content of the analyzed products was 19.8% (product B), representing 88% of total sugars determined, and this means 5.3 g/100 kcal; in this sample, the glucose was from the hydrolyzed flours. The glucose determined represents between 1.9% and 88.1% of total sugars, and the percentage of glucose was ≥50% of total sugars in half of the analyzed samples. The glucose content reported by crude flours is very low; previous studies performed on infant cereals showed a range of 0.03–0.09 g/100 g crude flours, and the hydrolysis with α-amylase produces an increase in glucose, maltose, maltotriose, and isomaltotriose [14]. In addition to hydrolysis, the addition of other ingredients such as malt extract, maltodextrins, fruit, and honey also influences the determined glucose content.

The percentage of fructose of total sugars was 0.4–15%; only in samples with honey and fruits, the percentage was above 2.8%. Some products added FOS, which did not contribute to the fructose contents of the products. Fernandez-Artigas et al. [14] determined significant contents of fructose and sucrose from banana and orange powders used as ingredients in infant cereals.

The percentages of sucrose of the total sugars contents in the samples with added sugars ranged between 27% and 61% and between 0.8% and 6.8% in samples without sucrose added; in the latter samples, the highest value is due to the addition of dehydrated fruits. The values of maltose were above 7.8 g/100 g in half of the samples. The percentage of maltose of the total sugars determined ranged between 7.7% and 95.6%. The last value corresponded to a sample with maltodextrin. Maltodextrins are added to infant food mainly to prevent caking, aid dispensability and solubility, provide texture to the products and reduce Maillard reactions.

Hydrolyzed maltodextrins, malt extract, and cereals have higher free sugars content, such as maltose and glucose. According to the label information, the flours from samples A–C, F, I, and K were enzymatically hydrolyzed, and the flours of sample J were thermally hydrolyzed during manufacturing. The rest of samples (D, E, G, H, and L) included, in the ingredients section label, “hydrolyzed” or “partially hydrolyzed” flours.

The average percentage of the sum of glucose and maltose of the total sugars content was 77.5%, and more than 40% of the samples analyzed had values higher than 95%; this content could vary considerably, if the hydrolysis conditions are not well established.

The ESPGHAN states that no sugar should be added to complementary foods, and free sugars should be minimized or avoided [28]. According to the mandatory nutritional information [29], the analyzed cereals with added sugars (sucrose) had values similar to products without added sugars. Samples A and B had a similar sugar content (22.5 g/100 g); sample A (“8 cereals and FOS”) was formulated with 87.7% of partially hydrolyzed cereals, sucrose, and FOS, and sample B (“8 cereals”), on the other brand, was formulated with 92% dextrinized cereal flours, FOS (3%), malt extract, and maltodextrin. They had a similar total sugars content, including one product with and the other without added sugars.

According to the Regulation (EU) 1169/2011 about the food information to consumers [29], the carbohydrate and sugar contents must be declared by law on the labels of all food products. It should be noted that, under the regulation, “sugars” are “all monosaccharides and disaccharides present in food”, meaning total sugars. For that reason, it is impossible to know if infant cereals subjected to the flour hydrolysis process comply with the legislation or exceed the levels of sugars allowed [25]. This is due to the impossibility of distinguishing the labeling between added sugars and intrinsic sugars of ingredients or these produced in the processing of porridge [30].

### 4.3. Sugar Content Determination by the Luff-Schoorl Method

The total sugars and sucrose contents were analyzed using the Luff-Schoorl method, commonly used for the determination of sugars on the label nutritional information. A high statistical correlation (r = 0.8455, *p* < 0.05) was obtained between both methods; however, both methods were statistically different (*p* < 0.05).

The total sugars content determined using the Luff-Schoorl method was higher than when using the HPLC method and closest to total sugars declared on the label. The Bland and Altman plot revealed good agreement between both measures (Luff-Schoorl and label); almost all points are scattered all over the place and between the two lines representing limits of agreement.

The regulation permits sugars tolerances of ±20% for sugar values between 10 and 40 g/100 g [29]. Eighty-three percentage of the samples were in these acceptability values by the volumetric method regarding the values indicated on the label. An extensive study of 28 food groups marketed in Spain (1173 products) showed 98% compliance with the EU tolerable sugar range [31], and a United States study (446 products) showed 63% compliance with the EU tolerable sugar range [32].

### 4.4. Estimated Energy Intake Provided by Sugars

Most commercial cereals provide 5% of daily calories from sugars with two feeds/day, with the average dose declared on the label being 33.2 g/feed of infant cereals. According to the results of this study, one of the main issues is the cereal amount per feed, being able to reach the recommended sugar limits depending on the dose used. Labeling should include the number of servings of the porridge per day according to the age and the sugar content of the porridge, to reduce the excessive added sugar intake [33,34,35]. However, the declared amount of sugars may vary from the actual amount due to permitted tolerances (± 20%). Foterek et al. showed that added sugar intake in infancy comes from complementary food, with that from complementary commercial foods being much higher than that from home-made foods [36]; however, recently the formulation of available commercial food products is changing.

According to the ENALIA survey [37] addressing children and adolescents in Spain in 2012–2014, the average total sugars intake (mono and disaccharides intrinsic or added sugar) was 95.1 g/day, being 21.5% of total calories. A survey in the US [35] evaluated the nutrient intake by ages between 2005 and 2012 and showed that the total sugars intake was 61.4 g/day for infants (0–6 months) and 92.2 g/day for children (12–24 months), similar to the results from the ENALIA survey.

The published literature on complementary feeding, especially for children below one year of age, concerning infant cereals is scarce. Infants are born with an innate preference for sweet taste, continuing in childhood and gradually waning in the adult stage [38]. However, it has not yet been possible to establish a clear association with their later food choices [39].

A theoretical sweetening power is shown in Table 5. The infant cereals with the lowest sweetening power are H and J, which had the lowest sugar content. Samples with a high sweetening power such as F and K had very different sucrose values, with 8.12% for cereals F and 0.18% for cereals K. Samples C and D with the highest values of sucrose were not the highest in sweetening power (1.36 and 1.51, respectively). Therefore, the sweetening power was not necessarily related to the added sucrose content. The addition of FOS only represented an increase of 0.1 in the value of the calculated sweetening power. There are brands of infant cereals analyzed that achieve a high sweetening power without added sucrose, and there are commercial infant cereals with a low proportion of sugars, but with sweetening power.

The relationship between the consumption of added sugars and possible negative health consequences is controversial. Some investigators claim that excessive sugar consumption is associated with an increased risk of caries [40], also influenced by the socioeconomic position and other lifestyle factors. Concerning obesity, there is no clear evidence that the alteration of the total carbohydrate proportion in the diet determines energy intake and consequently a higher body mass index [9]; a few studies have found a relationship for beverages and food with sugars [41]. Controversial results have been found regarding insulin resistance, glucose intolerance, and diabetes [39]. The EFSA issued a scientific opinion on the consumption of sugars and concluded that the risk of developing chronic metabolic diseases and cavities due to the fact that the ingestion of added sugars exists, and therefore, its consumption should be as low as possible; however, it has not been possible to establish a safe intake level of sugars [42].

#### Limitations and Strengths

Among the limitations, it should be taken into account that infant cereals are subjected to great innovations that involve the development of new formulations. Therefore, our study may not include commercial products currently available on the Spanish market. Besides, the sweetening power has been calculated theoretically, and no sensory analysis has been performed.

A noteworthy strength of this study is that the determination of the sugars contents by HPLC method allows knowing the content of sugars produced in the process and the content of sugars added to infant cereals.

## 5. Conclusions

Almost half of infant cereals have added sugars, with sucrose being the most common. A quarter of energy products come from sugars.

The European legislation of infant cereals has established individual values for added sugars, but the EU nutritional label information only exhibits the content of total sugars, but not that of added sugars. Moreover, the manufacture of infant cereals includes a starch hydrolysis stage, which provides a considerable and variable proportion of free sugars (glucose and maltose). The added sugars content should be included in the nutrition information on the label.

Even infant cereals without added sugars can have a high sweetening power due to the sugars obtained from hydrolysis.

## Figures and Tables

**Table 1:** Compositions (g/100 g) of infant cereal porridges obtained from the nutritional information listed on the label.

	Energy (kcal)	Fats (g)	SFA ^a^ (g)	Carbohydrates ^b^ (g)	Sugars (g)	Proteins (g)	Fiber ^c^ (g)
Mean ± standard deviation (SD)	382 ± 9	1.8 ± 1.1	0.42 ± 0.5	81 ± 4	23 ± 9	8.2 ± 1.9	4.7 ± 2.2
Median	380	1.5	0.30	81	25	8.5	4.7
Maximum	423	10	4.2	92	42	15	11
Minimum	362	0.70	0	67	0	3.9	0.60

^a^ Saturated fatty acid; ^b^ carbohydrate value does not include fiber content; ^c^ eight of 120 samples evaluated. Fiber was not included in the nutrition information.

**Table 2:** Numbers of products (infant cereals) with ingredients other than cereal flours.

Brands	Numbers of Products	Gluten-Free Infant Cereals (*n*)	Sucrose (*n*)	Honey (*n*)	Glucose (*n*)	Fruits ^a^ (*n*)	Cocoa (*n*)	Probiotics ^b^ (*n*)	Prebiotics ^c^ (*n*)	Others ^d^ (*n*)
B1	16	4	1	2	0	1	1	13	6	2
B2	10	3	10	1	2	2	0	0	3	0
B3	7	1	1	1	1	1	1	0	1	0
B4	2	1	0	0	0	0	0	0	0	1
B5	6	1	6	1	1	1	1	0	6	6
B6	22	4	10	6	0	4	1	0	2	6
B7	32	8	7	3	2	9	2	7	30	16
B8	9	4	9	2	0	1	0	0	4	3
B9	4	3	0	0	0	1	1	0	0	3
B10	4	1	4	1	1	0	0	0	3	3
B11	5	2	1	1	0	0	0	0	2	0
B12	3	1	3	1	3	0	0	0	2	1
Total (%)	120	33 (27.5%)	52 (43.3%)	19 (15.8%)	10 (8.3%)	20 (16.7%)	7 (5.8%)	20 (16.7%)	59 (49.1%)	41 (34.1%)

^a^ B1: prunes; B2: banana, apple, and orange; B3: banana, apple, and orange; B5: apricot, banana, and orange; B6: apple, pear, banana, orange, and pineapple; B7: orange, apple, banana, pineapple, pear, kiwi, grapes, and raisins; B8: banana, orange, and apple; B9: apple, pear, banana, and orange. ^b^ Probiotics: Bifidobacteria and Lactobacillus. ^c^ Prebiotics (soluble fiber): fructooligosaccharides, oligofructose, and inuline. ^d^ Other ingredients: skimmed yogurt, lime blossom extract, carob powder, malt extract, spelt, buckwheat, quinoa, tapioca, yucca starch, carrot, and nuts (almond, hazelnut, and walnut).

**Table 3:** Sugar contents (labeling information) and percentages of infant cereal porridges with added sugar, fruit, cocoa, and nuts.

Brands (*n*)	Mininum–Maximum Total Sugar Content (g/100 g)	Average Total Sugar Content (g/100 g)	Products with Added Sugar (%)	Products with Fruit, Cocoa, and Nuts (%)
B1 (16)	17.0–31.0	24.7	23.5	17.6
B2 (10)	0.2–41.7	24.8	100.0	16.7
B3 (7)	18.0–24.0	21.4	37.5	25.0
B4 (2)	0.3–1.0	0.7	0.0	0.0
B5 (6)	16.5–32.3	26.9	100.0	28.6
B6 (22)	3.2–39.4	21.8	50.0	27.3
B7 (32)	10.0–35.0	27.5	37.5	40.6
B8 (9)	20.4–32.3	23.3	100.0	10.0
B9 (4)	0.0–32.0	8.1	20.0	40.0
B10 (4)	20.4–24.0	22.6	100.0	0.0
B11 (5)	12.0–22.5	15.5	40.0	0.0
B12 (3)	26.1–40.4	35.5	100.0	0.0
Mean	-	23.9	54.3	38.6

**Table 4:** Sugar determination in infant cereal porridges (“8 infant cereals porridge”) of different brands by using the HPLC and Luff-Schoorl methods. Differences between both methods and differences between the total sugars contents determined and declared on the label.

	Sugars by HPLC						Sugars by the Luff-Schoorl Method				
Infant Cereals	Glucose (%)	Fructose (%)	Sucrose (%)	Maltose (%)	Total Sugars (%)	Difference from the Label (%)	Sucrose (%)	Total Sugars (%)	Difference from the Label (%)	Difference between Methods ^a^ (%)	Total Sugars Labeled (%)
A	1.21 ± 0.03	0.30 ± 0.02	6.09 ± 0.19	14.9 ± 0.53	22.5	−2.55	9.97	24.6	−0.38	2.17	25
B	19.8 ± 0.04	0.62 ± 0.02	0.32 ± 0.001	1.73 ± 0.07	22.5	−5.51	1.73	28.1	0.10	5.61	28
C	0.28 ± 0.009	0.36 ± 0.03	8.40 ± 0.54	5.76 ± 0.20	14.8	−7.80	9.14	20.4	−2.16	5.64	22.6
D	0.45 ± 0.01	0.35 ± 0.003	9.87 ± 0.17	5.42 ± 0.16	16.1	−6.41	13.7	21.5	−0.97	5.44	22.5
E	1.95 ± 0.009	0.95 ± 0.05	7.09 ± 0.07	12.9 ± 0.36	22.9	−4.45	10.3	26.8	−0.55	3.90	27.3
F	3.95 ± 0.14	0.34 ± 0.02	8.12 ± 0.18	9.95 ± 0.24	22.4	−7.14	13.6	27.5	−2.04	5.10	29.5
G	12.3 ± 0.13	0.10 ± 0.004	0.26 ± 0.015	1.83 ± 0.01	14.5	−6.48	0.64	21.4	0.43	6.91	21
H	6.19 ± 0.04	0.07 ± 0.0008	0.38 ± 0.003	2.24 ± 0.06	8.84	−3.12	1.24	15.3	3.29	6.45	12
I	9.55 ± 0.23	0.21 ± 0.001	0.37 ± 0.02	9.01 ± 0.17	19.1	−7.17	2.75	25.4	−0.90	6.27	26.3
J	0.46 ± 0.01	0.06 ± 0.002	0.15 ± 0.0004	14.4 ± 0.51	15.1	−5.09	0.30	12.5	−7.66	−2.57	20.2
K	11.3 ± 0.86	3.40 ± 0.11	0.18 ± 0.002	7.84 ± 0.58	22.7	−11.8	0.53	25.4	−9.07	2.72	34.5
L	11. 7 ± 0.53	1.14 ± 0.07	1.08 ± 0.03	2.07 ± 0.13	16.0	−6.04	2.89	21.0	−0.99	5.05	22

^a^ Differences between the total sugars contents by the using Luff-Schoorl and HPLC methods.

**Table 5:** Sucrose contents (g/100 g) and theoretical sweetener powers of infant cereals.

Infant Cereals	Sucrose (%)	Sweetener Power
A	6.09	1.72
B	0.32	1.77
C	8.40	1.36
D	9.87	1.51
E	7.09	1.85
F	8.12	1.86
G	0.26	1.11
H	0.38	0.67
I	0.37	1.36
J	0.15	0.94
K	0.18	1.83
L	1.08	1.31

## Data Availability

Data inquiries can be directed to the corresponding author.
